# Actual clinical leadership: a shadowing study of charge nurses and doctors on-call in the emergency department

**DOI:** 10.1186/s13049-018-0581-3

**Published:** 2019-01-08

**Authors:** Sissel Eikeland Husebø, Øystein Evjen Olsen

**Affiliations:** 1 Department of Quality and Health Technology, Faculty of Health Science, University of Stavanger, 4036 Stavanger, Norway; 2 Department of Surgery, Stavanger University Hospital, Stavanger, Norway; 3 Department of Research, Development and Education, Stavanger University Hospital, Stavanger, Norway; 4 Global Health Priorities Research Group, Center for International Health, Department of Global Public Health and Primary Care, University of Bergen, Bergen, Norway

**Keywords:** Charge nurses, Clinical leadership, Emergency department, Doctors on-call, Shadowing

## Abstract

**Background:**

The provision of safe, high quality healthcare in the Emergency Department (ED) requires frontline healthcare personnel with sufficient competence in clinical leadership. However, healthcare education curriculum infrequently features learning about clinical leadership, and there is an absence of experienced doctors and nurses as role models in EDs for younger and less experienced doctors and nurses. The purpose of this study was to explore the activities performed by clinical leaders and to identify similarities and differences between the activities performed by charge nurses and those performed by doctors on-call in the Emergency Department after completion of a Clinical Leadership course.

**Methods:**

A qualitative exploratory design was chosen. Nine clinical leaders in the ED were shadowed. The data were analyzed using a thematic analysis.

**Results:**

The analysis revealed seven themes: receiving an overview of the team and patients and planning the shift; ensuring resources; monitoring and ensuring appropriate patient flow; monitoring and securing information flow; securing patient care and treatment; securing and assuring the quality of diagnosis and treatment of patient; and securing the prioritization of patients. The last two themes were exclusive to doctors on-call, while the theme “securing patient care and treatment” was exclusive to charge nurses.

**Conclusions:**

Charge nurses and doctors on-call perform multitasking and complement each other as clinical leaders in the ED. The findings in this study provide new insights into how clinical leadership is performed by charge nurses and doctors on-call in the ED, but also the similarities and differences that exist in clinical leadership performance between the two professions. Clinical leadership is necessary to the provision of safe, high quality care and treatment for patients with acute health needs, as well as the coordination of healthcare services in the ED. More evaluation studies of this Clinical Leadership course would be valuable.

## Background

Hospital emergency departments (ED) play a vital role in the acute health care system, offering care for patients with acute illnesses and injuries and access to the health care system. The provision of safe, high quality healthcare in the ED requires frontline healthcare personnel with sufficient competence in clinical leadership (CL) [1–5]. However, healthcare education curriculum infrequently features learning about CL [6–8], and there is an absence of experienced doctors and nurses as role models in EDs for younger and less experienced doctors and nurses [6, 9]. Therefore, in 2013, the ED at Stavanger University Hospital (SUH) developed and implemented a clinical leadership course with the aim to reinforce core health system values and to foster an understanding of excellent day-to-day CL, and executing CL within a framework related to existing resources and organizational structure [10, 11]. This study is a part of a larger trailing research study on CL [11] where findings are systematically fed back to the course faculty to inform decisions on how to improve the CL course. Given the central role of CL in the ED, it is essential to describe participants’ activities after completion of the CL course to understand clinical leaders’ work activities, but also more specifically to increase understanding of how to improve the CL course to support the participants in achieving the course learning objectives. Additionally, it is expected that a deeper understanding of how healthcare personal perform CL activities will have the potential to improve awareness of current processes and inform education for future healthcare personnel.

In the existing literature, several definitions of CL have been formulated [1, 10, 12–14]. In conjunction with the CL course project, we redefined CL as to “take responsibility for clinical decision-making, within the scope of your role in a clinical team at any given time, with a patient-centred perspective addressing four key values: trust, quality, responsiveness, and efficiency” [10]. Several CL researchers have focused primarily on describing various attributes and characteristics of CL [15, 16] and behavioural-based competency models in CL programs [12], yet little scientific data exists to explore the performance of actual CL activities in the ED. Stanley [15] presented CL characteristics, including clinical expertise, involvement in care, a high level of interpersonal skills, the ability to act as role model, a commitment to high quality practice and empowerment of others. Internationally, studies in the ED setting have mostly focused on quantification of communication patterns [17–19], description of emergency nurses’ proactive activities as well as barriers to and opportunities for proactive work [20], and classification of the ED physician in charge’s problem solving actions [21]. These studies have revealed that providers in the ED have very high hourly task rates dominated by communication and clinical activities [18], and that staff members have to take control and make things happen, anticipating and preventing problems [20].

This study, however, did not focus on quantitative documentation of communication or activity among providers, but rather aimed to observe and describe the activities performed by clinical leaders, i.e. charge nurses (CN) and doctors on-call (DOC) in the ED after completion of the CL course. To address the aim of the study, the following research questions were formulated:

- What kind of activities do clinical leaders perform?
- What are the similarities and differences between the practice of CL when comparing the activities performed by CNs and those performed by DOCs?

## Methods

A qualitative exploratory design was chosen using shadowing and short conversations [22]. Shadowing is defined as a research technique that involves a researcher closely following a member of an organization over an extended period of time [23]. Shadowing was considered essential in gaining a full understanding of the type of activities clinical leaders performed in the ED, because of the ability to capture the brief, fragmented, varied, verbal and interrupted nature of organizational life, and it enables the identification of the context in which events happen [24]. More specifically, the primary advantage of shadowing is its mobility [25]. The target individuals in this study were shadowed (by SEH) from the moment they began their working day until their shift was finished. When appropriate, short conversations were elicited through questions asked by the researcher, and these questions received prompt responses or a running commentary from the person being shadowed.

### Participants

Five female CNs employed in the ED and three male and one female on-call doctors on rotation in the ED were shadowed. All DOCs were senior residents from the medical or the surgical departments. The nine participants met the study inclusion criteria as they had already completed a CL course, described below.

### Setting of the study

**Fig. 1:**
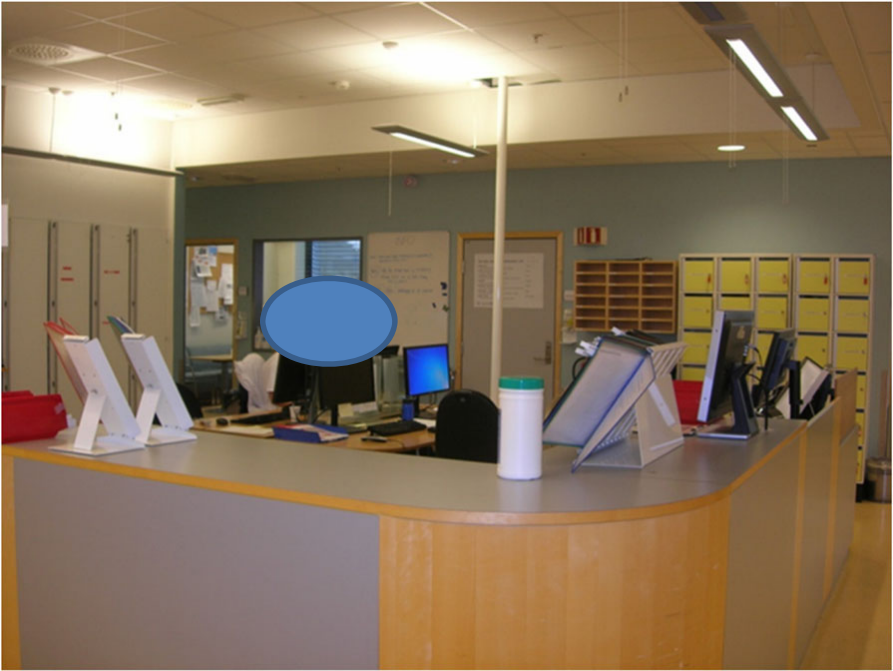
The central area of the treatment section

The ED at SUH is located in the south western part of Norway in an urban setting and triages approximately 30,000 patients per year. Every week emergency care is provided to 600 patients from 18 municipalities, from an overall population of about 350,000 people. The hospital is an academic teaching facility for nursing students from the University of Stavanger and medical students from the University of Bergen. The ED staff consists of 120 nurses, 40 attending physicians on rotation from medical, surgical and neurological departments, and various support personnel. Shadowing took place in the triage (14 beds), the treatment section (22 beds), the critical care area (4 beds) and the accident and the emergency surgical outpatient clinic (4 rooms). All providers, including CNs and DOCs who were shadowed, carried individually assigned portable wireless telephones to support communication. The central area of the treatment section houses several workstations equipped with computers, monitors, and telephones. The CN’s have access to a separate, dedicated space, and the same applies to DOCs (Fig. 1). The CN is expected to lead nursing and clinical support staff while managing the work systems and processes across the ED to ensure that the needs of patients are met. The CN is also involved in managing patient flow in collaboration with the DOC. The DOC’s role is to be responsible for patient triage, patient flow and the diagnostic and treatment plan of patients.

### The clinical leadership course

The vision of the CL course is to promote bedside values and an understanding of excellent day-to-day CL in teams, executing CL with existing resources and within the organisational structure. The development of the CL course followed the seven factors outlined by Salas et al. [26] and the didactic model of relation guided the design of the course [27]. The objectives of the course are to 1) function as skilled operative leaders and clinical supervisors within their everyday clinical setting, 2) understand and improve patient safety and quality, 3) understand the dynamics of patient flow, and critically and efficiently use available resources, and 4) improve trust between health personnel.

In developing the course content, both in materials and subject matter, five main contextualized topics were established: basics, behaviour, team, safety and tools. The course is structured in four steps comprising introduction, theory, workshop, simulation, and implementation in the workplace [10, 11]. The simulation scenarios focus on a patient with limited trauma and chest pain, lack of resources and overcrowding, prolonged length of stay in the ED, unclarified patients, bullying at work, and medication error with consequences. Pedagogical methods include workshops, simulation, group counselling and peer-to-peer dialogue, all of which emphasize guided reflection [28, 29]. The last two methods were chosen to support the implementation of CL and to facilitate the application of trained CL skills on the job [26].

### Data collection and analysis

Data collection took place shortly after the start of the CL course during November 2013 and March 2014. Data consisted of 70 h of field notes (40 pages) of participant observation (PO) (40 h of CNs and 30 h of DOCs) during day and evening shifts. During these times the treatment section was typically staffed with 1 to 2 attending senior residents, 2–3 residents, 1 CN and 10–14 nurses. Detailed notes were kept about who was being shadowed, the setting, day and time, roles, interactions and activities, including the running commentary from the person being shadowed.

The shadowing data were transcribed (by SEH) and then provided to the respective CNs and DOCs for comments or revisions to ensure clarity and accuracy. The first author then entered all transcripts into a Word document and printed these out in paper versions. The data were analyzed using a thematic analysis [30]. Initially, familiarity was established by reading and re-reading the data set. The transcribed text was broken down to meaning units. All meaning units were entered into a new Word document and printed out in paper versions. The Word document with all meaning units was divided into nine parts (nine observations) and attached to a wall. Small hand written Post-it in several colors were used to create preliminary codes (by SEH). The codes were refined by identifying similar observations or words. Codes that shared similar meaning were collated into categories (by SEH) by using Post-it. Themes were then formed by grouping categories and rechecked across the entire data. The themes were refined and discussed between the authors until consensus was reached. For examples of the analysis process, see Table 1.

**Table 1:** Examples of the analysis of the observations

Unit of meaning	Codes	Categories	Theme
The CN talks with the medical DOC, who says that medical patients have to wait for a long time. He ascertains that the level of activity is 2 according to the ‘Plan for High Activity’. The CN adds that it is quite full on the medical wards, but some beds are available on surgical wards. I think we have level 2 to 3.	Information on patient occupancy	Deciding activity level	1.Receiving an overview of the team and the patients and planning the shift
The DOC informs the CN that he is going to reallocate the medical doctors in the treatment section so that the patient followed by the police is quickly seen by a doctor.	Allocating doctors	Reallocating staff	2. Ensuring resources
The DOC tells the CN about two patients who can be transferred to the medical ward and a patient that can be discharged. In this regard he adds the following comment: “It is all about if we can have some advantages by ‘turning’ (early discharging) patients.	Providing patient discharge and patient transfer to medical units	Patient transfer	3. Monitoring and ensuring patient flow
The CN receives a call from the AMCC regarding a fire in a house downtown. The CN informs the DOC that doctors and anesthesiologists must not leave work. She tells the nurses and doctors in the treatment section to move all patients to units. She also informs a nurse to call for more nurses and the surgical DOC to empty the triage.	Instructing the staff to not leave work and transfer all patients to hospital units	Instructing in accordance with guidelines	4. Monitoring and securing information flow
The CN receives a fax from the AMCC about a patient with a cerebral injury. She calls the neurologist, asking if preparation should be made for actilysis treatment, CT scanning, and blood samples. She tells the nurse that a possible new actilysis patient will be placed in B3. The CN calls the radiology department and orders a CT scan and blood tests.	Activating the patient’s treatment team	Coordinating care	5. Securing patient care and treatment
The DOC and the medical student review the patient’s medical record, diagnosis and treatment. Further, the DOC guides the student regarding what to do and why and asks her to identify the relationships between symptoms and tests, further encouraging the student to consider this while he is looking at the ECG. Additionally, the DOC summarizes the patient’s health problems and identifies a future treatment plan. A little later, the DOC checks the patient’s medical record and then signs it.	Guiding and monitoring peers in diagnosis and treatment of patients	Supervising peers	6. Securing and assuring the quality of diagnosis and treatment of patients
The DOC tells the CN that the patient with high CRP must be prioritized into the treatment section. The DOC asks the junior doctor to have a look at the patient. The DOC looks at the medical record and examines the patient and then talks with a junior doctor about the patient’s diagnosis. The DOC tells the junior doctor to prioritize this patient.	Care of the patient	Seeing the patient	7. Securing the priority of patients

## Results

**Fig. 2:**
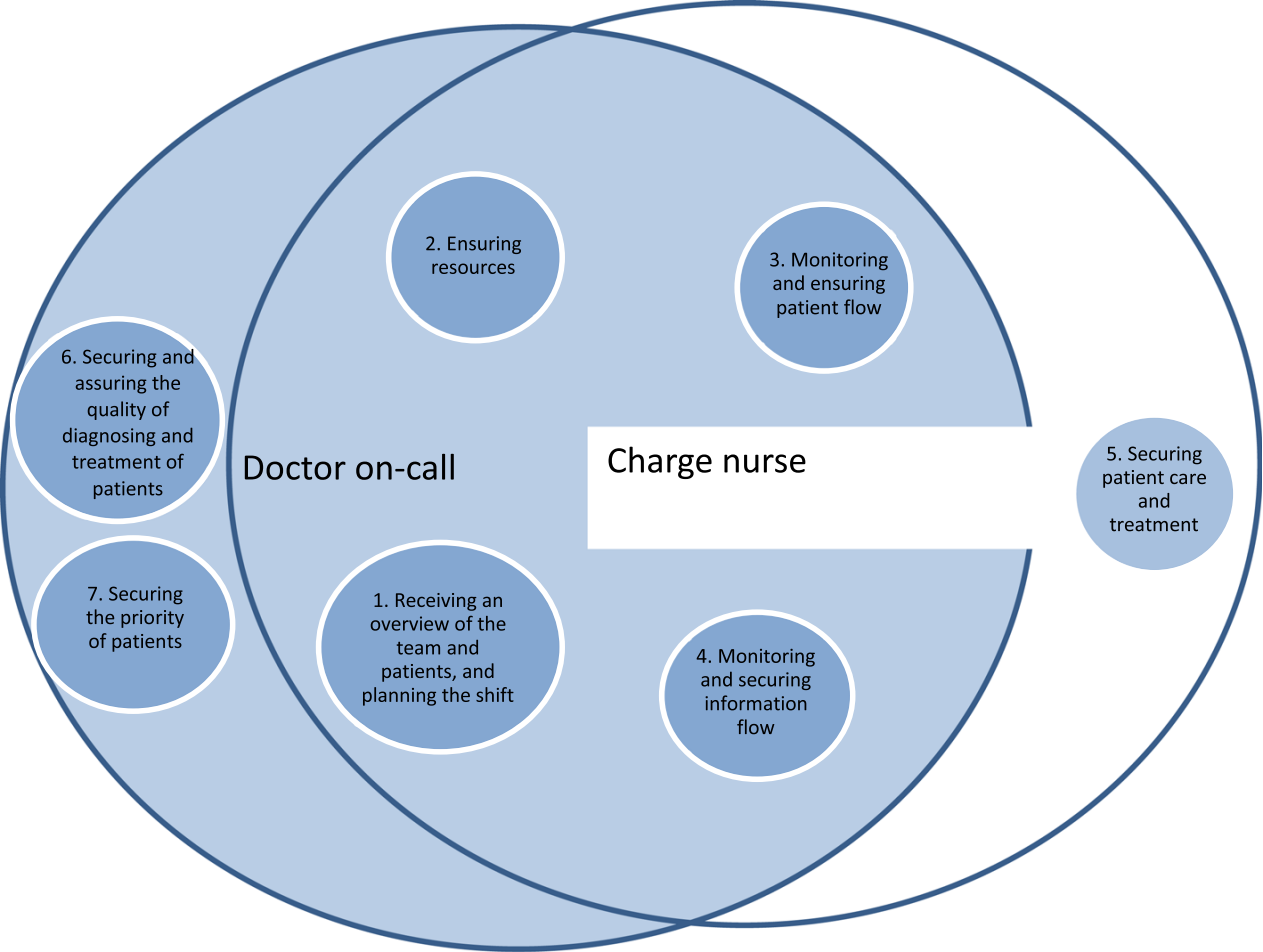
Themes regarding charge nurses and doctors on call as clinical leaders

The observations revealed that CNs and DOCs were simultaneously involved in a number of diverse activities pertaining to coordination and decision-making of patient treatment and care. Activities performed by CNs and DOCs were related to continuously monitoring the balance between patients, competence of staff and staffing numbers, and making adjustments to ensure quality of care, treatment and patient safety. Data revealed seven themes that were characteristic of the CNs’ and DOCs’ activities (Fig. 2): receiving an overview of the team and patients and planning the shift; ensuring resources; monitoring and ensuring patient flow; monitoring and securing information flow; securing patient care and treatment; securing and assuring the quality of diagnosis and treatment of patients; and securing the priority of patients. The last two themes were exclusive to DOCs, while the theme “securing patient care and treatment” was exclusive to CNs.

### Theme 1: Receiving an overview of the team and the patients and planning the shift

The observations revealed that the CN and DOC began each shift by collecting information regarding patient occupancy from the previous CN and DOC in a handover meeting. The CN allocated ED areas and tasks to nurses based on level of competence and number of nurses on the shift, and circulated these work distribution lists to triage staff, the accident and the emergency surgical outpatient clinic and treatment section. Allocation of staff, patient flow and occupancy were discussed between the DOC and the CN. Patient occupancy was registered in “Plan for High Activity” (Table 2), and the level of activity (rated from 0 to 3) was decided upon based on the criteria for each level, as illustrated in the following excerpts.

**Table 2:** Plan for High Activity

What is the problem?	Rating and Actions			
	Level 0	Level 1	Level 2	Level 3
Patients not being seen by a doctor within 1 h	< 6	6–11	11–20	> 20
Patients with a length of stay > 3 h	< 6	6–11	11–20	> 20
Total number of patients in ED	< 30	30–35	35–55	> 55
Identify cause	Monitor situation	Fill out Template for High Activity. Identify reason for delay in medical review and take action.	Revise the template and map again.	Revise the template.
A. Physician	Inform the senior resident when the number of medical patients ≥7. Inform the senior resident when the number of surgical/orthopedic patients ≥6.	Evaluate access to physicians, time spent, facilitate medical review. Inform the doctors on-call. Consider alerting the physician in triage in collaboration with doctors on call.	Evaluate access to physicians, time spent, facilitate for medical assistance. Inform the doctors on-call. Consider preparing your physician in triage in collaboration with doctors on call.	Ask for telephone meeting through AMCC. Report on: 1.Number of patients. 2.Causes 3. Actions already implemented
B. Patient flow 1. Allocation to units.		Check the patient flow procedure. Inform the patient flow coordinator about the level of activity.	Inform the patient flow coordinator about the level of activity. Assist doctors on-call for reviewing patients code green in the triage area, and if necessary prior to triage. Further diagnosis and treatment takes place in the ward.	
2. Length of stay > 3 h.		Inform and discuss with responsible radiographer/biochemist about level of activity and coordination of actions.	Inform and discuss with responsible radiographer/biochemist about level of activity and coordination of actions. Evaluate if patients are transferred directly to the units before examinations and blood samples.	
3. Lack of infection room/ telemetry services in the units.		Discuss with the patient flow coordinator. Coordinate with the doctor on-call about reprioritizing.	Discuss with the patient flow coordinator. Discuss with the doctor on-call. Contact the service centre for infections for quick de-contamination of rooms.	
C. Location		Use waiting space 1–5 to release treatment beds. Use the ED critical care area; re-triage to treat, transfer or discharge patients.	Move patients to the corridor to release treatment beds. Use “The accident and the emergency surgical outpatient clinic” in cooperation with staff.	
D. Nurse				
1. Long time as “ready for the doctor”.		Consider reallocating nurses to triage for physician assistance.	Consider reallocating nurse to triage for quicker patient flow. In collaboration with doctors on-call, consider reallocating patients from the observation unit to “The accident and the emergency surgical outpatient clinic”.	
2. Long time as “ready for transfer to unit”.		Reallocate nurses from other areas of the ED.	Reallocate nurses to the ED in cooperation with the Observation unit and “The accident and the emergency surgical outpatient clinic”.	
3. Too few nurses		Authority to call nurses into work up to a total of 9 nurses in the ED.	Authority to call nurses into work up to a total of 9 nurses in the ED.	

*AMCC* Acute Medical Communication Central, *ED* Emergency Department

*“The CN talks with the medical DOC, who says that medical patients have to wait for a long time. He ascertains that the level of activity is 2 according to the ‘Plan for High Activity’. The CN adds that it is quite full on the medical wards, but some beds are available on surgical wards. I think we have level 2 to 3.” (PO 3).*

The “Plan for High Activity” includes initiating special interventions on each level, which is on the agenda in the next excerpts:

*“The DOC discusses the level of activity with the CN. They agree on level 3, because there is no room available for a single patient and there are few available beds in the medical wards. They discuss whether a medical patient should be transferred to a surgical ward and check the number of discharge patients during the day.”(PO 7).*

While the DOC checks the patient flow database, he provides the following commentary:

*“It is important to check the yellow (triage code yellow) patients in triage who are lying for many hours. That is why I regularly check in the patient flow database.” (PO 8).*

### Theme 2: Ensuring resources

From the observations it was evident that both the CN and the DOC continuously ensured the proper allocation of staff for patient care and treatment so that all patients received a responsible nurse and doctor. The former ensured the appropriate number of nurses for patient care, and the latter ensured the appropriate allocation of doctors for patient diagnostics and treatment, which is illustrated in the following excerpts:

*“The CN receives a call from the triage area regarding more staff. Thereafter, she asks a nurse to go to triage and help.* “*(PO 3).*

*“The DOC informs the CN that he is going to reallocate the medical doctors in the treatment section so that the patient followed by the police is quickly seen by a doctor” .(PO 7).*

### Theme 3: Monitoring and ensuring patient flow

From the observations, it was noted that the CNs and DOCs communicated frequently regarding patient flow, whether it was patient transfers from the triage to the treatment section or from the ED to the other wards. They often monitored the number of patients triaged in the ED patient flow database, which is illustrated in the next excerpts:

*“The DOC asks the CN if a patient can be transferred from triage to room 13 in the treatment section. The CN calls the triage nurse and asks if she can transfer the patient into room 13. The CN then walks to the break room and tells the nurse that a patient is expected in room 13.” (PO 2).*

*“The DOC tells the CN about two patients who can be transferred to the medical ward and a patient that can be discharged. In this regard he adds the following comment: “It is all about if we can have some advantages by ‘turning’ (early discharging) patients.” (PO 6).*

Sometimes, the DOC first collected information from the junior doctor regarding patients who were ready to be transferred:

*“The DOC tells the CN that more medical patients should be transferred from the triage to the treatment section. Two rooms are available, and we will put medical patients in there, he says. The DOC asks the junior doctor if some of the patients can be moved to the corridor, and he responds by identifying these patients. The DOC tells the CN which patients can be moved to the corridor.” (PO 7).*

### Theme 4: Monitoring and securing information flow

The CN and DOC have key roles in the disaster emergency plan for the ED. In one of the shadowing observations, a deviant situation arose. There was an alarm from the Acute Medical Communication Central (AMCC) regarding a fire downtown. The alarm led to several interventions in the ED, i.e. providing updated information to all key personnel and to the head of department, as illustrated below:

*“The CN receives a call from the AMCC regarding a fire in a house downtown. The nurse on duty informs the DOC that doctors and anesthesiologists must not leave work. She tells the nurses and doctors in the treatment section to move all patients to units. She also informs a nurse to call for more nurses and the surgical DOC to empty the triage.” (PO 4).*

Another important task for the CN and DOC that the observations revealed was to provide guidance and information to new doctors and nurses regarding how the patient flow database and the electronic patient record work. Additionally, they guided new peers in procedures, record systems and medical equipment, as illustrated here:

*“The CN informs the new junior doctor how the patient flow database is working and about the contamination routines. The CN tells the nurse and doctor not to take the bladder scanner into a contaminated room.” (PO 1).*

*“The DOC guides new peers regarding how the electronic medical record is working and how new blood tests can be ordered electronically.” (PO 6).*

### Theme 5: Securing patient care and treatment

This theme only emerged during observations of CNs. Unlike the DOCs, the CNs coordinated care and treatment for admission of new patients immediately after a request from either the DOC, the triage nurse, the ambulance services or the AMCC. The CN also provided necessary medical equipment for patient treatment, as exemplified below:

*“The CN receives a fax from the AMCC about a patient with a cerebral injury. She calls the neurologist, asking if preparation should be made for actilysis treatment, CT scanning, and blood samples. She tells the nurse that a possible new actilysis patient will be placed in B3. The CN calls the radiology department and orders a CT scan and blood tests.” (PO 2).*

### Theme 6: Securing and assuring the quality of diagnosis and treatment of patients

The observations showed that the DOC was continuously “hands on” regarding the diagnosis and treatment of patients. The orthopedic DOC also functioned as the team leader in trauma situations. The DOC conducted quality assurance of junior doctors and medical students, and made decisions about further plans for diagnosis and treatment of patients, as illustrated below:

*“The DOC and the medical student review the patient’s medical record, diagnosis and treatment. Further, the DOC guides the student regarding what to do and why and asks her to identify the relationships between symptoms and tests, further encouraging the student to consider this while he is looking at the ECG. Additionally, the DOC summarizes the patient’s health problems and identifies a future treatment plan. A little later, the DOC checks the patient’s medical record and then signs it.” (PO 7).*

According to the “Trauma team” guidelines, the orthopedic DOC assumes the role of the team leader when a “code red” trauma patient is admitted to the ED. In one of the observations the team leader informed the team about clinical findings and gave directives to the team members about the treatment of a “code red” trauma patient:

*“The orthopedic DOC states that the patient has decreased respirations on the left side. A team member reposts vital signs and respiration rate. The team leader states: “No injury after fracture, one iv access so far, and saturation 80. Can you find heat sheets? How are the pupils? A nurse reads the results of the blood gas: normal findings. The DOC states that the ear temperature is 36.5 C, and that the staff cut off the patient’s clothes.” (PO 9).*

### Theme 7: Securing the priority of patients

This theme only emerged during the shadowing of DOCs. DOCs delegated to junior doctors which patients should receive medical attention first. DOCs also informed the CN which patients in the triage should be prioritized and transferred to the treatment section, as illustrated in the following:

*“The DOC tells the CN that the patient with high CRP must be prioritized into the treatment section. The DOC asks the junior doctor to have a look at the patient. The DOC looks at the medical record and examines the patient and then talks with a junior doctor about the patient’s diagnosis. The DOC tells the junior doctor to prioritize this patient.” (PO 6).*

## Discussion

The findings demonstrate that the clinical leaders perform multitasking by constantly monitoring patient and information flow, allocation of resources, patient safety and quality of care. What distinguishes our findings from previous studies is how the CNs and DOCs complement each other as clinical leaders by continuously striking a balance between the bedside operational values (quality, efficiency, interprofessional trust and responsiveness) [cf. 10].

Similar to the attributes of clinical leaders outlined in Daly et al. [1], we found that both CNs and DOCs were directly involved in patient care, although the doctors were more hands-on in the diagnosis and treatment phase than the CNs. The findings demonstrate that clinical leaders provided the essential resources of knowledge and experience for less experienced peers. Daly et al. [1] noted similar findings in their review of major attributes of CL.

Both CNs and DOCs were occupied with receiving an overview at the start of and during the shift. This skill is one of nine core skills in Emergency Medicine [31] associated with patient safety in the ED [31], and it is an essential factor in the U.S.- based TeamSTEPPS program [32].

Findings also reveal that the clinical leaders were constantly monitoring the allocation of resources (efficiency) to secure quality of care and safety for patients, and to accommodate patient expectations (responsiveness). These skills, except the latter, are components of the “Clinical leadership checklist” self-assessment tool outlined by McSherry and Pearce [4]. Similar to our findings, CL commitment has been found to be a key factor in improving patient flow in the ED [33–35]. For example, in a study conducted by Zocchi et al. [35], leadership was found to be one of the fundamental elements in improving ED flow measures in hospitals. We found that the CNs and the DOCs play a vital role in maintaining information flow within their own and across other professions in the ED, and this finding is congruent with the results of a previous study that reviewed effective communication in CL [16]. Similar to the study findings, Pun et al. [36, 37] found that information was repeated several times. We found some differences between CL activities performed by CNs and DOCs. These findings can be explained by the differences in responsibilities between the medical and nursing professions. The main duties of a doctor are prevention of disease and diagnosis and treatment of patients [38]. In the ED setting, doctors need additional competencies, including prioritization of patients [39], leadership and team leadership, decision making, effective communication and collaboration [8]. Nurses’ competencies for example, comprise conducting nursing assessment and planning for and providing safe and effective nursing care [40]. Working as a frontline CN requires additional qualities, such as management of communication, team coaching, and serving as a role model [41].

A limitation of the study concerns that only nine CLs in one ED were shadowed. The small sample size of CNs and DOCs would undoubtedly introduce some selection bias into the study. To achieve validity, we have tried to make the data collection and analysis process as transparent as possible. Additionally, the presence of a researcher taking notes may have changed the CNs and/or DOCs practice behavior.

However, the findings in the current study provide new insight into how CL is performed by CNs and DOCs in the ED, and how they complement each other as clinical leaders. The findings have several implications for CL course development and practice. First, managers can employ the insights into the activities of the CNs and DOCs to emphasize and guide important aspects of CL in daily work and to monitor and evaluate operations in the ED. Second, clinical leaders can utilize the findings to enhance their competence in CL. Finally, the content of CL courses can be improved by paying attention to the findings from this study.

## Conclusion

CNs and DOCs perform multitasking and complement each other as clinical leaders in the ED to secure safe, high quality patient care. The findings from this study provide new insights into how CL is performed by CNs and DOCs in the ED, but also the similarities and differences that exist in CL performance between the two professions. CL is necessary to the provision of safe, high quality care and treatment for patients with acute health needs, as well as the coordination of healthcare services in the ED. More evaluation studies of this CL course would be valuable.

## Abbreviations

CL: Clinical Leadership
CN: charge nurse
DOC: doctor on-call
ED: Emergency Department
SUH: Stavanger University Hospital
